# How object naming dissociates from repetition and comprehension impairments when post stroke aphasia is less severe

**DOI:** 10.1038/s41598-026-41575-3

**Published:** 2026-03-14

**Authors:** Storm Anderson, Rachel M. Bruce, Thomas M. H. Hope, Sophie M. Roberts, Kate Ledingham, Hayley Woodgate, Jennifer T. Crinion, Alexander P. Leff, David W. Green, Cathy J. Price

**Affiliations:** 1 Department of Imaging Neuroscience, University College London, London, UK; 2 Institute of Cognitive Neuroscience, University College London, London, UK; 3 Department of Brain Repair and Rehabilitation, University College London, London, UK; 4 Department of Experimental Psychology, University College London, London, UK

**Keywords:** Anomia, Aphasia, Incidence, PPV, Sensitivity, Stroke, Diseases, Medical research, Neurology, Neuroscience

## Abstract

Object naming is widely used for assessing aphasia. We provide the first quantitative analysis of how well: (A) impaired spoken object naming (anomia) detects auditory repetition and/or speech comprehension impairments and (B) intact naming rules these impairments out. Participants were 382 stroke survivors (1 month to 34 years post-stroke) with impaired naming, repetition and/or comprehension, but intact object recognition. We assessed: (1) Incidence of anomia within the full sample; (2) its positive predictive value (PPV), i.e., the proportion of patients with anomia who had impaired repetition and/or comprehension; (3) its sensitivity to other impairments, i.e., the proportion of patients with impaired repetition or comprehension who had anomia; and (4) how object naming, word repetition, sentence repetition, word comprehension and sentence comprehension compared in their incidence, PPV and sensitivity, when each was treated as the reference task. Incidence, PPV and Sensitivity of anomia were 66%, 90% and 63% across sample; 93%, 100% and 93% for the most severely aphasic patients and 50%, 86% and 46% for the remaining patients. These metrics were not higher for object naming than sentence comprehension, sentence repetition and word repetition; but word comprehension showed markedly lower incidence and sensitivity. Although anomia may be the most salient symptom of aphasia in everyday conversation, our findings (i) challenge assumptions that object naming is a superior test of aphasia, (ii) show that the presence of anomia was insensitive to 37% of patients with repetition and comprehension impairments and (iii) highlight how PPV and sensitivity within an aphasic sample are influenced by impairment severity, task dependency, measurement variability and inter-patient differences.

## Introduction

Word finding difficulty (anomia) is considered to be a hallmark indicator of aphasia^1–4^. Prior studies have described anomia as: “the most common deficit present across different aphasia syndromes” [^4^, p. 3042#1:2]; “the most frequent and pervasive symptom for people with aphasia, regardless of the aphasia type” [^1^, p. 1#1:3]; “the most common symptom of aphasia post-stroke, often regardless of severity and lesion location” [^3^, p. 3040#1:2]; “the cardinal deficit of people with aphasia” [^5^, p. 364#1:1]; “present in practically every aphasic patient” [^2^, p. 1#3:2], and “ranks among the most noticeable and universal of aphasic characteristics” [^6^, p. 229#1: 1].

As a result, object naming is widely used in both clinical and research assessments of aphasia, for example the Boston Diagnostic Aphasia Examination [BDAE^7^], the Western Aphasia Battery [WAB^8^], the Minnesota Test for Differential Diagnosis of Aphasia [MTDDA^9^], the Porch Index of Communicative Ability [PICA^10^] and the Comprehensive Aphasia Test [CAT^11^]. It is typically assessed in aphasia testing both post-stroke^5,12,13^ and in neurosurgical assessments^14,15^. Indeed, there are many practical and clinical advantages of including object naming in aphasia assessments. For example, it is well-validated, reliable, time-efficient, easy to administer, easy to score, and can be done at the bedside or in any other patient-care setting^16,17^. Object naming errors also provide insight into the integrity of speech production and the language system as a whole and can be used to inform areas that need further assessment^18,19^. Despite the prominence of object naming in both clinical assessment and aphasia studies, most prior work on the co-occurrence of different impairments has examined *continuous relationships* between task scores, using regression or correlation analyses that estimate how variations, across individuals, in performance on one task relates to variation in performance on other tests. For example, several studies^17,18,20,21^ have shown a strong correlation between object naming scores and overall aphasia severity measured using the Western Aphasia Battery^8^. This result is informative but does not distinguish patients with “co-occurring” or “selective” impairments (i.e., where one ability is preserved while another is impaired). In contrast, “incidence” classifies performance categorically (impaired vs. intact) to quantify patterns of overlap and dissociation among impairments within an aphasic sample, thereby offering novel insights into the clinical and cognitive structure of aphasia. For example, Hybbinette et al.^22^ found that impaired word repetition, as measured by the Apraxia of Speech Rating Scale^23^ was prevalent in 80% (12/15) of their patients with “aphasia”, according to the Neurolinguistic Aphasia Examination^24^. In an extensive review of the aphasia literature, we only identified three reports^25–27^ of the incidence of naming impairments (anomia). Incidence in this study refers to the proportion of patients within the study sample who present with a given impairment (e.g., anomia) at the time of testing, which is equivalent to the base rate of that impairment in the dataset. More generally, incidence can also refer to the rate at which new cases of a condition occur over time within a defined population; however, in the present cross-sectional research study context, it is used to describe the observed prevalence of impairments in the tested cohort. The first study^25^ we identified reported the relative frequency of different symptoms according to a questionnaire, but provided few methodological details. The second study by Richardson and colleagues^26^ investigated the relationship between naming and functional communication. In addition, the incidence of anomia within their cohort of patients with aphasia, defined by the Western Aphasia Battery [WAB^8^], can also be derived from their published figures, using the Boston Naming Test cut-offs provided by Abeare et al.^28^. This showed that 191 of 233 patients (82%) classified as aphasic on the WAB had naming impairments on the Boston Naming Test. The third quantitative study by Grönberg and colleagues^27^ was more specific - it assessed 58 aphasic patients who had impaired performance on at least one of four language tasks (naming, word repetition, sentence repetition, word comprehension and sentence comprehension) in the acute stage after stroke (2–6 days), and found that the incidence of anomia was highest (79%), followed by impaired sentence comprehension (64%), word and sentence repetition (62%) and word comprehension (41%).

Critically however, the task with the highest incidence of impairments does not necessarily correspond to the task that shows greatest overlap with other impairments. For example, patients with selective impairments in lexical retrieval may increase the number of individuals classified as having anomia without affecting repetition or spoken comprehension performance because lexical retrieval is essential for object naming but not for repetition or spoken comprehension. Additionally, Grönberg et al.’s^27^ finding that only 79% of patients with impairments on at least one of their language tasks (naming, repetition or comprehension) had anomia indicates that 21% of patients with aphasic repetition and/or comprehension did not have anomia. Likewise, 18% of patients classified as having aphasia by the Western Aphasia Battery in Richardson et al.^26^ did not show anomia.

To our knowledge, there are no published studies that quantitatively examine how well the presence or absence of anomia is associated with the presence or absence of impairments on other language tasks. Typically, prior studies refer to review papers^29,30^ that make incidence claims without quantitative evidence and are more concerned with whether object naming ability detects (i) neurocognitive disorders^31^; (ii) seizure location in epilepsy^32,33^, (iii) Alzheimer’s Disease^34^ and (iv) conversational/connected speech ability^5,12,16^.

The primary objective of the current study was to deepen our understanding of the relationship between post-stroke anomia and impaired repetition and comprehension, within a large, chronic post-stroke aphasia cohort. “Impaired” tasks were defined according to the aphasic cut-off scores in the Comprehensive Aphasia Test [CAT^11^; see Methods section]. Unlike the Richardson et al.^26^ study that measured the incidence of anomia in patients with any type of aphasia (including fluency and sentence completion as well as repetition and comprehension), we focus on the five core language tasks selected by Grönberg and collegues^27^: spoken object naming, auditory word repetition, auditory sentence repetition, spoken word comprehension and spoken sentence comprehension. These tasks are particularly relevant because they are widely used in aphasia batteries and screening tools such as the National Institutes of Health Stroke Scale [NIHSS^35^], the Aphasia Rapid Test [ART^36^] and the Quick Aphasia Battery [QAB^37^]. However, to explore the effects of task and participant selection, we also report whether and how the results change when adding additional tasks, such as reading. Finally, in line with the Grönberg et al. study^27^, we refer to anomia as impairments in spoken object naming, but we also test how the results would change if we opened up the definition of anomia to include impairments on action naming.

There are three main differences between the study by Grönberg et al.^27^ and the current one. First, unlike Grönberg et al.^27^ who reported the incidence of anomia in the first few days after stroke, the current investigation reports the incidence of anomia in the chronic stage (months to years) post-stroke. The overlap between language task impairments in the chronic stage post-stroke may differ from the acute post-stroke stage, measured by Grönberg et al.^27^, because different language processes may recover at different rates, resulting in improved performance on some tasks while other tasks remain more challenging. Indeed, the profile of language impairments has already been reported to be different in the late versus early post-stroke phase. For example, Global aphasia in the first week post stroke typically evolves into less severe syndromes within the first year post stroke^38,39^. The increased severity of aphasia symptoms in the acute stage post-stroke^40^ may be due to physiological factors such as neuronal inflammation and oedema^41^, with subsequent recovery involving reperfusion of ischemic penumbra^42^, functional reorganization and repair^43,44^ and psychological factors that exacerbate language performance such as fatigue and low mood^45^.

Second, to advance our understanding of patterns of co-occurrence among language impairments, the current study also applies two conditional measures commonly used in medical research: “positive predictive value” and “sensitivity”^46^. Grönberg et al.^27^ used these measures to examine the diagnostic accuracy of the NIHSS test. Here we use positive predictive value and sensitivity to characterise associations between anomia and other language impairments within a large aphasic cohort (*n* = 382), rather than to establish diagnostic accuracy in distinguishing aphasia from non-aphasia.

The *positive predictive value* of anomia indicates the proportion of patients with anomia who also had impaired repetition and/or comprehension. If most patients with anomia have impairments in other language tasks, then anomia has a high positive predictive value. On the other hand, if few patients with anomia have other language impairments, then anomia has low positive predictive value. In contrast, the *sensitivity* of anomia for detecting other types of language impairments was assessed as the proportion of patients with impaired repetition or comprehension who also had anomia. This differs from the *incidence* of anomia which was assessed as the proportion of the total sample (*N* = 382) who had anomia (rather than the proportion of patients with aphasic repetition or comprehension who had anomia). Although sensitivity is expected to increase as the incidence of an impairment increases, sensitivity—not incidence—depends on factors like (i) how related the tasks are in terms of cognitive processing and mental effort, (ii) whether the tasks are supported by the same brain structures, and (iii) whether the patient has a large lesion that impairs multiple brain regions supporting multiple tasks. If tasks are similar or use the same resources, impairments on one task will impair performance on other tasks. Likewise, patients with large lesions are more likely to have impairments on multiple tasks.

Knowing the sensitivity of anomia informs how the absence of a naming impairment rules out the presence of impairment on other tasks. For example, if all patients with impaired comprehension exhibit anomia, then the absence of anomia would indicate the absence of a comprehension impairment, eliminating the need to formally assess comprehension abilities. However, if many patients with comprehension difficulties show no signs of anomia, then only testing object naming would miss patients with comprehension impairments.

The third difference from the Grönberg et al.^27^ study is that we assessed how the incidence, positive predictive value and sensitivity of anomia vary as a function of (i) the severity of anomia and the severity of repetition and/or comprehension impairments; (ii) patient selection and (iii) task selection. An effect of aphasia severity has previously been observed when considering the incidence of apraxic speech. Specifically, 100% (7/7) of patients with “severe aphasia” [defined by the Neurolinguistic Aphasia Examination^24^] also had apraxic speech; whereas this dropped to 63% (5/8) in those with less severe aphasia^22^. Theoretically, an uncoupling between performance on different tasks is more likely when scores are in the mild range because of ambiguity in the threshold for indicating aphasic performance. This ambiguity arises from wide inter-patient variability in pre-stroke language skills. For instance, someone whose pre-stroke naming abilities fall in the low normal range (e.g., 5th percentile of a normative cohort) may be misclassified as having a “selective” post-stroke anomia when formally assessed if post-stroke performance is relatively better on repetition and comprehension tasks than the naming task. Likewise, a person could also be misclassified with selective impairments if (i) they were fatigued and lost concentration specifically during that test, or (ii) they had sensory, perceptual or object recognition problems that impacted performance on one task more than others^47–49^.

The effect of task selection was investigated from four perspectives. First, we compared how the positive predictive value and sensitivity of object naming varied for each repetition and comprehension task separately. Second, we investigated how the results changed when patients with “reading” and “semantic picture matching” impairments were also included in our sample. Third, we investigated the effect of broadening our definition of anomia to include patients who had action naming impairments. Fourth, we compared how positive predictive value and sensitivity changed when the “reference task” was systematically changed from object naming to word repetition, sentence repetition, sentence comprehension and word comprehension. In so doing, we address the potential limitation that, because object naming only assesses language at the single word level, it may not be sensitive to sentence level impairments.

From a clinical perspective, investigating patterns of co-occurrence among impairments on different language tasks provides a quantitative overview of how anomia and other common language impairments co-exist in the chronic phase post-stroke within aphasic populations. This has not been empirically investigated before and requires a large cohort of stroke survivors whose language abilities were assessed months to years post-stroke, using the same standardised tests.

Finally, this study’s data-driven, quantitative analysis of the relationship between post-stroke anomia and other language impairments provides a methodological framework for future studies examining relationships among impairments in aphasia, which are in turn important for looking to develop diagnostic aphasia tools that are sensitive to detecting aphasia, regardless of language tasks or severity.

## Methods

### Patient selection

All patients were selected from the Predicting Language Outcome and Recovery After Stroke (PLORAS) database, which comprises extensive demographic, behavioural and neuroimaging data from a large sample of stroke survivors^50^. All participants gave written informed consent prior to participation and were compensated £10 per hour for their time. The study was approved by the London Queen Square Research Ethics Committee (13/LO/1515) and conducted in accordance with the Declaration of Helsinki.

A total of 382 patients with left hemisphere stroke damage (including those with bilateral damage) were selected. The majority of the patients (*n* = 363; 95%) were in the chronic post-stroke phase (6 months to 34 years), with the remaining patients (*n* = 19) ranging from 1 to 5 months post stroke. All had aphasic performance on at least one of the following five tasks from the Comprehensive Aphasia Test [CAT^11^]: spoken object naming, auditory word repetition, auditory sentence repetition, spoken word comprehension and spoken sentence comprehension; with no missing data for any of these five tasks. The aphasia cut-offs in the CAT were established, by the CAT authors, using data from 27 neurotypical controls. The lowest 5% of control scores were classified as “aphasic.” This control group covered the age and educational ranges of the aphasic patient group but with proportionally more women.

Additional inclusion criteria ensured that all 382 patients: were native English speakers, born in the United Kingdom to minimize the influence of any cultural or dialect differences, and self-reported that they had intact/or corrected vision and hearing. Finally, 49 patients with impaired semantic picture matching (as tested by the CAT semantic memory test) were excluded from the main results to ensure that the analyses focused specifically on language processing impairments, rather than deficits in object recognition, semantic association, or comprehension of task instructions. As reported in the Results, however, the study conclusions were minimally impacted by the inclusion or exclusion of these 49 patients.

Demographic details of the participants are provided in Table 1.

**Table 1:** Demographic data of the included patients.

Demographic and clinical details			*N* = 382
Age at stroke (years)		Mean (SD)	**55.6** (13.6)
		Range	17.2–85.9
Age at time of language test (years)		Mean (SD)	**61.1** (12.9)
		Range	19.2–87.5
Years since stroke		Mean (SD)	**5.6** (5.7)
		Range	0.1–34.3
Years of formal education (from 14 years of age)*		N with 0–1 years	19
		N with 2 years	173
		N with 3–4 years	48
		N with 5–7 years	69
		N with 8 + years	64
Total lesion volume (cm^3^)	Left hemisphere	Mean (SD)	**91.7** (101.7)
		Range	< 1–1025.5
	Right hemisphere	Mean (SD)	**7.0** (56.6)
		Range	< 1–1088.7
Gender		N female	114
		N male	268
Object naming raw/T-scores Impaired ≤ 43/≤ 61		Mean (SD)	**34** (15)/**57.8** (9.9)
		Range	0–48/37–74

Object naming scores were calculated according to the Comprehensive Aphasia Test manual, which also specifies the impairment threshold. SD, standard deviation; hem, hemisphere. Mean values are highlighted bold. *missing education data for 9 included patients.

### CAT T-scores

To enable comparison across CAT language tasks, the CAT manual recommends that raw scores are converted to clinical test norming T-scores^11^. These express relative performance within a reference sample on a scale constructed to follow a normal distribution but differ from statistical T-scores which are derived via linear standardisation. In contrast, clinical test norming T-scores for clinically heterogeneous populations are based on nonlinear procedures to enable scores from subtests with differing distributional properties to be compared on a common scale.

A three-step norming procedure, specified by the test authors^11^, is used to calculate CAT T-scores. First, raw scores for each subtest are converted to percentile ranks based on an aphasic reference sample^11^. This transformation is non-linear because percentile ranks are distribution-dependent, such that equal differences in raw scores do not correspond to equal differences in percentile rank. Nevertheless, the relative standing of a score within the aphasic reference sample is preserved and this relative position is prioritised over absolute raw-score differences. The aphasic reference sample, used in the standardisation of the CAT^11^ included 113 individuals with 266 total test scores, as 56 were re-tested. The inclusion of repeated tests reflects a norming approach designed for clinical standardisation rather than population-level inference. In patient cohorts, such as those with aphasia, where recruitment is challenging and performance distributions are often sparse or highly skewed, repeated assessments can improve the stability of norm-referenced scoring and incorporate clinically relevant variability over time, albeit at the expense of the independence assumptions typically associated with percentile estimation. The CAT also uses test scores from 27 neurotypical controls, after transforming them to the same aphasic reference sample which ensures consistent norm-referencing and alignment of percentile-based cut-offs across groups.

In the second step, the percentile ranks were transformed into Z-scores using the properties of a standard normal distribution (i.e. via an inverse-normal probit transformation). This non-linearly maps relative standing onto an approximately normal metric, allowing scores from subtests with different distributional properties to be compared on a common scale and normally distributed within the aphasic population.

In the third step, the Z-scores were converted into T-scores using the standard equation: T = 10Z + 50, yielding norm-referenced scores with a mean of 50 and a standard deviation of 10 in the normalised reference distribution^51^. In our sample of 382 patients, the mean/SD T-scores for each task were: 52.4/7.1 (cut-off = 61) for naming, 48.5/5.7 (cut-off = 56) for word repetition, 47.7/6.5 (cut-off = 56) for sentence repetition, 53.4/6.1 (cut-off = 60) for sentence comprehension and 46.9/4.2 (cut-off = 51) for word comprehension.

### Five CAT tasks of interest

#### Spoken object naming

Patients were instructed to name aloud 24 objects (e.g., knife, spoon), presented one at a time as black and white line drawings. The items vary in word length, frequency, morphological complexity, regularity, imageability (the extent to which the stimuli evoke a clear mental image) and animacy (i.e., animate vs. inanimate), which are all known to impact naming performance^52–54^. However, other variables, known to affect naming^53,54^ are not explicitly controlled in the CAT, such as age of acquisition, phonetic (or phonological) complexity, semantic category, and prototypicality. Spoken object naming is marked out of 48 points across 24 items (2 points per item). Correct answers are given a score of 2 per item if the name was produced within 5 seconds and a score of 1 per item if the correct name was produced after 5 seconds of the item being shown (a delayed response). Incorrect answers are given a score of 0, as are omissions, and phonemic, semantic, neologistic and dyspraxic errors. Dysarthric speech errors are not penalized (i.e., distortion of phoneme) as long as the perceptual identity of the target word is unaffected. If the patient self-corrected an error without an external cue, a score of 1 is given. The score remains at zero if the patient only produced a correct response after an external phonemic or semantic cue. A T-score of ≤ 61 (raw score of 43/48) was the aphasic cut-off (i.e., indicated an impaired performance within the CAT’s aphasic range).

#### Auditory word repetition

Patients were asked to repeat a total of 16 heard words that varied from high to low imageability, low to high frequency-use and mono to multi-syllables (e.g., vine, president, scorn). A T-score of ≤ 56 (raw score of 29/32) was the aphasic cut-off.

#### Auditory sentence repetition

Patients were asked to repeat spoken sentences (e.g., “the cat chased the bird”) which are grouped into 4 levels of difficulty, measured by the number of content words per sentence. If a sentence is repeated correctly, the patient moves to the next level of sentence repetition, and if not repeated correctly, the patient is afforded another opportunity to repeat a sentence at the same level. A T-score of ≤ 56 (raw score of 10/12) was the aphasic cut-off.

#### Spoken word comprehension

On each of 15 trials, patients heard object names and were visually presented with four pictures. They were asked to choose (by pointing or selecting with the computer mouse) the picture that matched the target item (e.g. goat). The three other pictures were distractors, which were semantically related (e.g. sheep), phonologically related (e.g. coat) and unrelated (e.g. dress). A T-score of ≤ 51 (raw score of 25/30) was the aphasic cut-off.

#### Spoken sentence comprehension

The task was similar to the spoken word comprehension task, but the target stimuli were 16 heard sentences (i.e., “the dancer paints the policeman”) and the distractors were pictures showing the same lexical items in different syntactic structures (i.e. a picture of a *policeman* painting a *dancer*), and different lexical items in the same syntactic structure (i.e. a picture of a dancer *chasing* the policeman). A T-score of ≤ 60 (raw score of 27/32) was the aphasic cut-off.

### Three additional CAT tasks used to assess the influence of task selection

#### Semantic memory

Patients were asked to choose (by pointing/selecting with the computer mouse) a correct semantically related picture (e.g., an eye) to the target item (e.g., reading glasses) in the presence of three other semantic and unrelated distractors (e.g., ear, mouth, elephant). This subtest is a version of the widely used Pyramids and Palm Tree test^55^. A T-score of ≤ 47 (raw score 8/10) was the aphasic cut-off.

#### Action naming

Patients were instructed to name the action, depicted in black and white line drawing pictures. There are a total of five test trials plus a practice trial at the beginning. We interpret these scores with caution^56–58^, particularly because the actions that patients were required to name in the CAT are arguably less routine nowadays (i.e., “typing” on an old-fashioned typewriter, “winding” a watch, “licking” a stamp, “threading” a needle) thereby increasing the risk of picture-misinterpretation and false positive results. Articulatory errors (e.g., dysarthric distortions) not affecting the perceptual identity of the target word are scored as correct responses. Verbal, phonemic, neologistic and apraxic errors are scored as incorrect responses. A T-score of ≤ 59 (raw score 8/10) was the aphasic cut-off.

#### Overall reading aloud

Patients were asked to read 24 words (e.g. *family*), 3 complex words (e.g. *informative*), 3 function words (e.g. *but*) and 5 nonwords (e.g. *fask*). Performance across all four reading tasks was reflected by a summary T-score, as specified in the CAT. As with naming, articulatory errors (e.g., dysarthric distortions) not affecting the perceptual identity of the target word are scored as correct responses. Verbal, phonemic, neologistic and apraxic errors are scored as incorrect responses. A T-score of ≤ 60 (raw score 62/70) was the aphasic cut-off.

### Data analysis steps

To calculate positive predictive value (PPV) and sensitivity, anomia and other language impairments were treated as binary categorical variables (i.e., impaired/intact) in line with conventional reporting. A limitation to this approach is that it doesn’t distinguish between different levels of naming performance and therefore gives equivalent weight to patients with the most and least severe naming impairments within the cohort.

To explore the potential influence of severity on our metrics, we treated severity categorically because its distribution within task and across patients was non-normal. As the CAT does not provide criterion-referenced severity cut-offs (e.g. mild, moderate, severe) or direct measures of functional communication, we define severity relative to the position of the patients within the score distribution of our aphasic sample, rather than clinically defined severity or functional communication status at the population level. In other words, CAT scores are used to characterise relative differences in language performance within the large cohort of patients with aphasia (*n* = 382) tested here, which provided the full range of possible aphasic scores for each of the spoken tasks (object naming, word repetition and sentence repetition).

“Most severe aphasia” was defined, on a task-by-task basis, as the range of scores for the third of patients with aphasic scores that were in the lowest range on that task. All other scores in the aphasic range were referred to as “Less severe aphasia”. Within the Less Severe group, the “Least severe” aphasia was defined as the range of scores for the third of patients with aphasia who had the highest scores in the aphasic range, see Table 2 for details.

McNemar’s test was used to compare paired categorical outcomes across tasks and severity. When reporting the incidence, PPV and sensitivity of Anomia in detecting aphasic performance on any of the repetition and comprehension tasks (Results Sections  1–3), we compare “Most-Severe” and “Less-severe” (including all those who were not in the Most Severe range). When reporting PPV and Sensitivity for each task pair (Results Section  4), we compare Most Severe and Least Severe (i.e., the third lowest scores and the third highest scores within the aphasia range).

**Table 2:** Definition of most and least severe aphasic performance on each task.

Participants with impairments	Most severe scores				Least severe scores			
	*N*	%	Raw	T	*N*	%	Raw	T
251 Object naming	80	32	0–21	37–49	87	35	38–43	58–61
220 Word repetition	74	34	0–15	35–45	77	35	26–27	52–56
227 Sentence repetition	71	31	0–2	39–42	86	38	8–10	53–56
256 Sentence comprehension	84	33	0–20	28–52	94	37	25–27	57–60
76 Word comprehension	25	33	0–22	32–46	21	28	25	51

Left panel shows the number of participants with each impairment. Middle panel shows the number and proportion of participants with the most severe impairments (raw = raw score range; T = T-score range). Right panel shows the same for the participants with the least severe impairments. Details for the participants with intermediate scores are not shown but can be deduced. The proportions in the Most and Least severe aphasia ranges are not exactly 33% because multiple patients shared the same scores at the range boundaries. Maximum raw scores for each task are 48 (Object naming), 32 (Word repetition), 12 (Sentence repetition), 32 (Sentence comprehension) and 30 (Word comprehension).

### Calculating incidence, PPV and sensitivity for anomia

Incidence, positive predictive value (PPV) and sensitivity were calculated for each task using a 2 × 2 contingency table (Table 3). Incidence refers to the proportion of patients within the sample who present with a given impairment (e.g., anomia) at the time of testing, equivalent to the base rate of that impairment. PPV represents the proportion of patients impaired on the reference task (cells C + S in Table 3) who have co-occurring impairments on other tasks (cell C). We use PPV descriptively to quantify patterns of symptom co-occurrence among individuals with aphasia, rather than as a measure of diagnostic accuracy. Sensitivity reflects the proportion of patients impaired on the other tasks (cells C + N) who have co-occurring impairments on the reference task (cell C). We use sensitivity as a conditional probability of co-occurring impairments within an aphasic sample, rather than diagnostic sensitivity relative to an external reference standard. Note, the numerator in both the PPV and sensitivity equations is always C (co-occurring impairments). The only difference in the equations is whether the denominator includes: S (selective impairments on the reference task) or N (no impairment on the reference task). A high proportion of selective impairments on the reference task will reduce PPV, whereas a high proportion of selective impairments on the other tasks (cell N) will reduce sensitivity. In addition, because PPV and sensitivity each express the conditional probability of one impairment given the presence of the other, they would take on identical values if “aphasia on repetition and comprehension” were treated as the reference task and anomia as the task to be detected.

Finally, we define the anomia absence rate as 1 − Sensitivity (see Table 3) which reflects the proportion of patients without anomia among those impaired on other language tasks. This approach avoids reliance on conventional negative predictive value (NPV) or specificity, which (i) require inclusion of individuals without aphasia and estimation of true negatives, and (ii) are not possible to estimate in our current study because all patients had aphasia on at least one of the five CAT language tasks. Likewise, agreement metrics (e.g. overall accuracy) are not reported because the study does not include a non-aphasic comparison group against which correct rejection could be defined. By focusing on Incidence, PPV and Sensitivity, we provide a detailed characterisation of how language impairments co-occur across tasks and severities within aphasia, offering quantitative insight into the informativeness of commonly used language measures in patients with chronic post-stroke aphasia.

**Table 3:** Calculation of incidence, PPV and sensitivity for anomia.

Anomia?	Repetition or comprehension impaired?	
	Yes	No
Yes	Co-occurring (*n* = 227)	Selective anomia (*n* = 24)
No	No anomia (*n* = 131)	OK in all (*n* = 0)

Metric	Definition/formula	Value
Incidence	(C + S)/(C + S + N)	251/382 = 66%
PPV	C/(C + S)	227/251 = 90%
Sensitivity	C/(C + N)	227/358 = 63%
Anomia absent rate:	1 – Sensitivity	1–63% = 37%
Selective anomia	S/(C + S + N)	24/382 = 6%

C = anomia with co-occurring impairments (on comprehension and/or repetition tasks).

S = selective anomia which means that the patients did not have aphasic performance on any of the other four study tasks but such ascription does not exclude impairments on tasks that are not considered in this study (e.g. spoken picture description). N = No anomia (but by selection all these patients will have repetition and/or comprehension impairments). OK = intact across all tasks (these patients were deliberately excluded from the analysis, therefore, in the current study, this number is always zero). Number in brackets = total number of patients in each cell. Anomia absent rate = 1 − sensitivity (diagnostically known as false negative rate) which indicates what proportion of patients with other impairments do not have impairments on the reference task.

### The incidence of anomia: How frequently is anomia observed in post-stroke aphasia?

As in Grönberg and colleagues’^27^ study, the incidence of anomia was calculated as the proportion of patients with anomia (as measured by the object naming task) within all patients who had impaired scores, at or below the aphasia cut-off levels on any of the 5 tasks described above. Incidence was calculated for (i) the whole sample, (ii) the sample reduced to patients with the lowest (most severe) scores and (ii) the remaining sample with less severe scores.

### Positive predictive values (PPVs): How frequently is anomia associated with impaired repetition and comprehension?

Positive predictive value (PPV) was calculated as the proportion (%) of patients with anomia (*N* = 251) who had co-occurring deficits on repetition and/or comprehension, see Table 3. To assess the effect of severity, PPV was calculated (i) across all patients with anomia, (ii) within the subgroup of patients with the most severe anomia, and (iii) within the subgroup of remaining patients with less severe anomia. In all analyses, PPV quantified the co-occurrence of anomia with repetition and comprehension impairments irrespective of the severity of those repetition or comprehension impairments. To assess how co-occurrence varied by language domain, these analyses were repeated for each task separately (i.e. word repetition, sentence repetition, word comprehension and sentence comprehension).

### Sensitivity: How frequently is anomia observed in patients with impaired repetition and/or comprehension?

The sensitivity of anomia was calculated as the proportion (%) of patients with impaired repetition and/or comprehension (*N* = 358) who also had anomia, see Table 3. To assess the effect of severity, sensitivity was calculated (i) across all patients with impaired repetition and/or comprehension, (ii) within the subgroup of patients with the most severe repetition or comprehension impairments, and (iii) within the subgroup of patients with less severe repetition or comprehension impairments. In all analyses, sensitivity quantified the co-occurrence of anomia (1) across anomia severity (i.e., irrespective of severity) and (2) for those with the most severe anomia. To assess how sensitivity varied by language domain, these analyses were repeated for each task separately (i.e. word repetition, sentence repetition, word comprehension and sentence comprehension).

### Which task is most strongly associated with other impairments?

In the analyses of incidence, PPV and sensitivity above, Object naming is treated as the “reference task” meaning that the presence of impairments on other language tasks is evaluated conditional on impaired naming performance. In Sect.  4, we report how incidence, PPV and sensitivity change when each of the “other tasks” (i.e., word and sentence repetition and comprehension) are treated as the “reference task” and object naming is one of the “other tasks”. For example, the sensitivity of word repetition to anomia was the proportion of patients with anomia (*N* = 251) who also had impaired word repetition; while the sensitivity of sentence comprehension to word repetition was the proportion of patients with impaired word repetition (*N* = 220) who also had impaired sentence comprehension.

### How PPV and sensitivity change with task selection

Three additional CAT tasks were included in an exploratory add-on analysis to assess the influence of task selection on PPV and sensitivity. Specifically, the incidence and PPV of object naming were compared when (i) the 49 patients with semantic matching impairments were included or excluded from the analyses; (ii) patients with impaired action naming, but not object naming, are treated as anomic; (iii) reading impairments are also considered.

## Results

### The incidence of anomia: How frequently is anomia observed in post-stroke aphasia?

The most frequently observed impairment in the full sample of 382 stroke patients with CAT scores in the aphasic range, was sentence comprehension (67%) followed by anomia (66%), sentence repetition (59%), word repetition (58%), and word comprehension (20%).

When the sample was reduced to patients with the lowest scores within the cohort (i.e. most severe impairments) on at least one of the five tasks (*n* = 138), incidence was again highest for sentence comprehension and anomia (93%) followed by sentence repetition (92%), word repetition (87%) and word comprehension (36%). In the remaining 244 patients (with less severe impairments), incidence fell to 52% for sentence comprehension, 50% for anomia, 41% for word and sentence repetition and 11% for word comprehension.

### Positive predictive value (PPV): How frequently does anomia co-occur with impaired repetition and/or comprehension?

Within the sample of 251 patients with anomia, 90% were impaired on at least one of the repetition or comprehension tasks, with the remaining 10% having “selective” naming impairments (preserved repetition and comprehension). Critically, however, PPV of anomia depended on which language tasks were being considered and the severity of anomia.

Across anomia severity, PPV of anomia was 67%, 71% and 75% for word repetition, sentence repetition, and sentence comprehension respectively, but fell to 25% for word comprehension. The drop in PPV of anomia for detecting word comprehension impairments can be explained by the lower incidence of word comprehension compared to the other tasks. Specifically, because the incidence of word comprehension (20%) was less than a third of the incidence of anomia (66%), the maximum achievable PPV of anomia for detecting word comprehension impairments is approximately 30%.

**Fig. 1:**
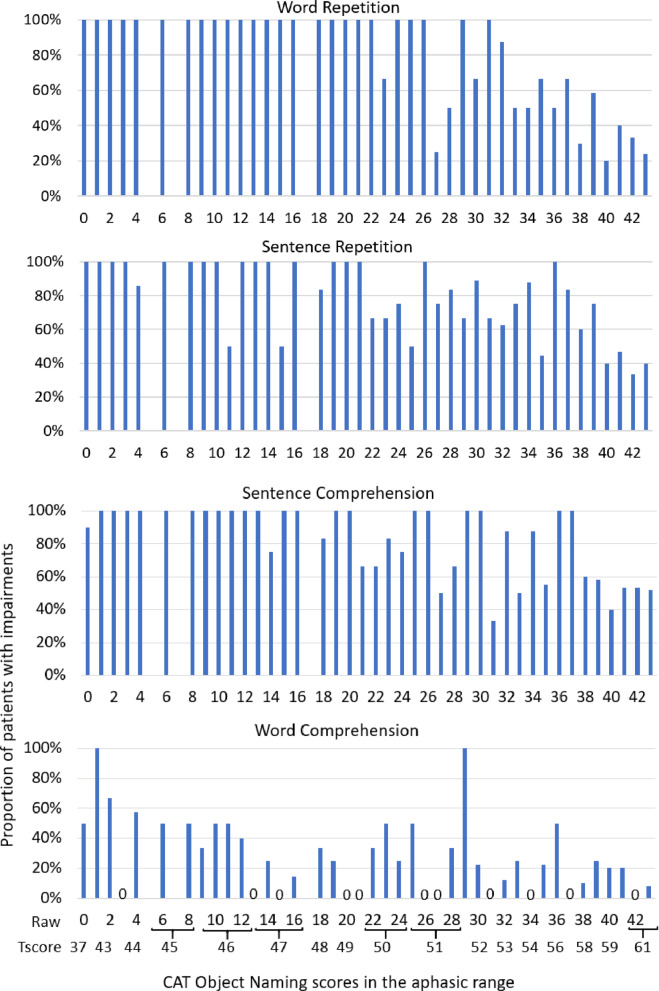
PPV: Proportion of patients with anomia (*n* = 251) who also had impaired repetition and/or comprehension, plotted against each object naming score in the anomic range. These figures plot the proportion (%) of patients with impairments on each repetition or comprehension task, on the y axis, against object naming raw scores, in the anomic range (0–43), on the x axis. Corresponding T-scores are included in the final plot for information. Note: empty columns indicate the associated naming scores were not observed in current cohort (i.e., raw naming scores 5, 7, 17). Columns replaced with “0” indicate that none of the patients with those naming scores had co-occurring word comprehension impairments.

For the 80 patients with the most severe anomia (defined in Table 2): PPV of anomia was 100% for word repetition; 95% for sentence repetition, 94% for sentence comprehension; and 39% for word comprehension, see Fig. 1.

For the remaining 171 patients with less severe anomia, PPV was 86% across tasks. For individual tasks, PPV fell to: 51% for word repetition, 60% for sentence repetition; 66% for sentence comprehension; and only 18% for word comprehension, see Fig. 1.

### Sensitivity: How frequently is anomia observed in patients with impaired repetition and/or comprehension?

**Fig. 2:**
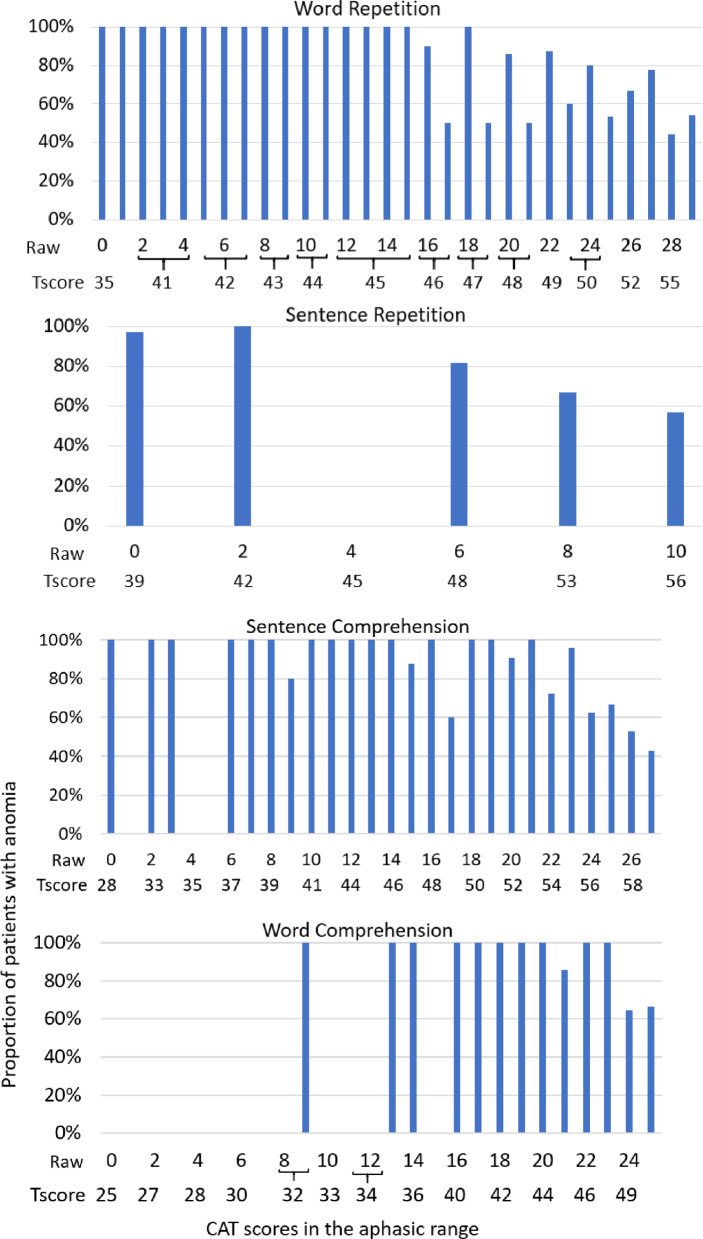
Sensitivity: Proportion of patients with anomia for each of the other repetition and comprehension scores in the impaired range. The proportion of patients in the anomic range, on the y axis, is plotted against each repetition or comprehension task raw and T-scores, in the impaired range, on the x axis. n = number of patients with scores in the aphasic range for each repetition and comprehension task. Note: empty columns indicate the associated scores were not observed in current cohort. There were no instances where there were repetition or comprehension scores that were never associated with object naming (i.e. like those labelled 0 in Fig. 1).

Within the 358 patients with impaired repetition or comprehension, only 227/358 (63%) had anomia. Anomia was therefore absent in 131 (37%) of the patients with impaired repetition or comprehension, indicating that the absence of anomia does not reliably imply the absence of aphasia on any other task. However, sensitivity of anomia was significantly higher for patients with more severe than less severe repetition or comprehension impairments, (Odd’s ratio = 15.7; *p* < 0.0001), as detailed below and in Fig. 2.

Among the 131 patients with the most severe repetition or comprehension impairments, 122 (93%) had impaired object naming; with 56% having the most severe anomia. These numbers were in a similar range when detecting impairments on each task separately: 100%/78% of patients with the most severe word repetition impairments had anomia/the most severe anomia, 97%/72% for sentence repetition, 92%/51% for sentence comprehension and 96%/72% for word comprehension. Given that the most severely impaired repetition or comprehension was strongly associated with anomia (> 90%), the absence of anomia is strongly associated with the absence of the most severe repetition or comprehension impairments.

In the remaining 227 patients with less severe repetition or comprehension impairments, 46% had anomia, and 1% had the most severe anomia. Again, these numbers were in a similar range when detecting individual impairments: 55%/7% for word repetition impairments, 62%/6% for sentence repetition, 56%/5% for sentence comprehension and 59%/7% for word comprehension. Thus, the absence of the most severe repetition or comprehension impairments is associated with a very low likelihood of the most severe anomia (< 10%).

**Fig. 3:**
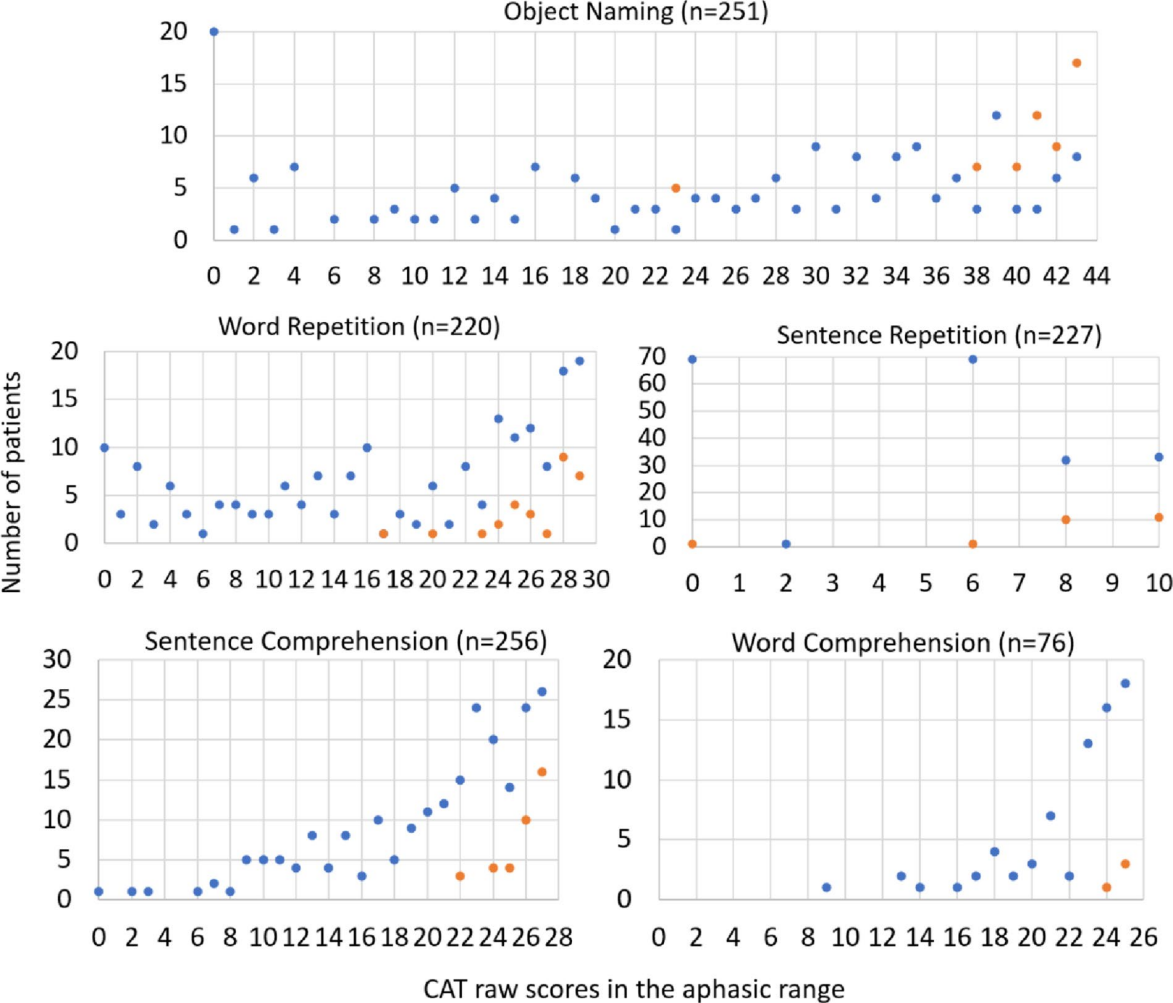
An illustration that selective impairments, on each task, are typically in the least severe range. This figure illustrates that, for each task, selective impairments (orange dots) are typically in patients with the least severe aphasia. The x axis shows the CAT raw scores in the aphasic range (lower = more severe). The y axis shows the number of patients who have each score. Blue dots indicate patients with non-selective impairments (i.e., impairments that co-occur with impairments on other tasks).

The effect of severity on the sensitivity of anomia arises because all but one of the 93 patients with “selective” impairments on repetition or comprehension had the least severe aphasic scores (as defined in Table 2). In contrast, only one patient with selective impairments had the most severe level of impairment (on sentence repetition), see Fig. 3. Note that “selective impairments” are defined as aphasic on only one of the five tasks in this study. This definition precludes co-occurring impairments with object naming, thereby reducing both sensitivity and PPV (see Table 3) but it does not preclude impairments on other language measures such as spoken picture description that were not included in this analysis.

### Which task is most strongly associated with other impairments?

Word comprehension was the least informative reference task (see Table 4), with very low sensitivity regardless of severity (13–42%), reflecting the low incidence of word-comprehension impairments in our sample (20%) compared with the other tasks (58–67%). However, in terms of positive predictive value, when word-comprehension impairments were present, they frequently co-occurred with other impairments: 84% had sentence-comprehension impairments and 82% had naming impairments.

The other four tasks showed broadly similar patterns of co-occurrence, with the relative strength of the association varying slightly depending on which task was the reference task and which impairment was being detected. For example, sentence-comprehension impairments were detected in 77% of patients with sentence-repetition impairments and 75% of those with naming impairments, whereas naming impairments were detected in 79% of patients with sentence-repetition impairments and 73% of those with sentence-comprehension impairments.

### How the PPV and sensitivity change with task selection

If we additionally include action naming in the task selection, and consider anomia as impaired object or action naming (rather than only considering object naming), the incidence of anomia rises from 66% to 80% but critically PPV of anomia falls from 90% to 78%. These changes occur because there were 52 patients with aphasic action naming who did not have impairments on any of our 5 tasks (i.e., would be reflected in cell S in Table 3 if included).

If we additionally include reading in the task selection, the incidence of anomia falls from 66% to 63% (because there were 15 patients with reading impairments who did not have impairments on any of our 5 tasks), with a small rise in PPV from 90% to 93%.

If the 49 patients with impaired semantic memory had been additionally included, the incidence of anomia would have risen from 66% to 69% and the incidence of sentence comprehension would have risen from 67% to 70%.

These results do not change any of our conclusions but highlight that the quantification of PPV and sensitivity varies with the task selection.

**Table 4:** Comparing language tasks in relation to co-occurring impairments.

Reference tasks	PPV for each other task					Sensitivity for each other task				
Across severity level	ON	WR	SR	SC	WC	ON	WR	SR	SC	WC
251 impaired ON	**~**	67%	71%	75%	25%	~	76%	79%	73%	82%
220 impaired WR	76%	**~**	68%	73%	25%	67%	~	66%	63%	74%
227 impaired SR	79%	66%	**~**	77%	24%	71%	68%	~	68%	71%
256 impaired SC	73%	63%	68%	**~**	25%	75%	73%	77%	~	84%
76 impaired WC	**82%**	**74%**	71%	**84%**	**~**	25%	25%	24%	25%	~

**Most severe aphasia**										
80 impaired ON	**~**	**100%**	95%	94%	39%	~	100%	97%	92%	96%
74 impaired WR	**100%**	**~**	91%	89%	35%	100%	~	92%	83%	96%
71 impaired SR	97%	92%	**~**	93%	30%	95%	91%	~	98%	88%
84 impaired SC	92%	83%	**98%**	**~**	42%	94%	89%	93%	~	100%
25 impaired WC	96%	96%	88%	**100%**	**~**	39%	35%	30%	42%	~

**Least severe aphasia**										
87 impaired ON	**~**	33%	47%	53%	13%	~	56%	62%	51%	67%
77 impaired WR	56%	**~**	47%	53%	16%	33%	~	47%	46%	43%
86 impaired SR	62%	**47%**	**~**	53%	17%	47%	47%	~	38%	48%
94 impaired SC	51%	46%	38%	**~**	15%	53%	53%	53%	~	57%
21 impaired WC	**67%**	43%	**48%**	**57%**	**~**	13%	16%	17%	15%	~

The left column lists each reference task and the number of patients impaired on that task. The middle panel shows the positive predictive value (PPV), i.e., the proportion of patients impaired on the reference task who were also impaired on each other task. The right panel shows sensitivity, i.e., the proportion of patients impaired on each other task who were also impaired on the reference task. Because PPV and sensitivity each express the conditional probability of one impairment given the presence of the other, sensitivity values are the transpose of the corresponding PPV values. PPV and sensitivity are shown separately for the full sample and for most severe and least severe aphasia subgroups. ON, Object naming; WR, Word repetition; SR, Sentence repetition; SC, Sentence comprehension; WC, Word comprehension. The highest value in each column is Bolded to identify the most effective reference task for detecting other impairments. WC shows the highest PPV but the lowest sensitivity, reflecting the low incidence of WC (20%) within the sample. ON, WR, SR, and SC are broadly similar.

## Discussion

Object naming is widely regarded as “the most common symptom of aphasia post-stroke”^1,3,4^ and is often treated as a highly sensitive indicator of language impairment^59^. By investigating symptom co-occurrence within a large group of patients with aphasia, the current study’s findings add important nuance to these claims by showing that, in a large cohort of patients with chronic post-stroke aphasia, the incidence of anomia was only 66%. This means that testing object naming alone failed to capture approximately 37% of patients with repetition or comprehension impairments, and provided limited information about the broader pattern of language difficulties.

A second striking finding was that impairments in sentence comprehension, sentence repetition and word repetition showed levels of overlap with other impairments that were comparable to anomia, indicating that naming is not uniquely informative. Plausibly, the prominence given to object naming in both clinical and research settings is that word-finding difficulties are particularly salient in everyday communication because they disrupt speech flow and produce overt errors, whereas repetition and comprehension impairments may remain less apparent unless directly assessed.

A third finding was that the extent to which any task provided information about other impairments depended strongly on the severity of impairment in the reference task. When impairments on the reference task were severe, co-occurrence with other impairments was high, with PPV and sensitivity approaching ceiling (> 90%) across naming, repetition and sentence comprehension tasks. In contrast, when patients had the least severe impairments on the reference task, PPV and sensitivity of these tasks fell to 12–67%. The most severe impairments on the reference task were therefore far more informative about impairments on other tasks than the least severe impairments on the reference task.

If we now consider the anomia absent rate (1-sensitivity), we see that the absence of anomia (as measured by the CAT Object Naming task) is (i) strongly associated with the absence of the most severe repetition or comprehension impairments (which almost always co-occurred with anomia), but (ii) not necessarily associated with the absence of less severe repetition or comprehension impairments. From a practical perspective, this means that preserved object naming substantially reduces the likelihood of severe repetition or comprehension impairments, but does not reliably rule out the presence of less severe repetition or comprehension impairments.

A fourth finding was that, despite the high overlap in naming, sentence comprehension, sentence repetition and word repetition impairments, these impairments less frequently co-occurred with word comprehension impairments. This result is likely related to the lower incidence of word comprehension impairments in our aphasic sample (20%) compared to the other four tasks (58–67%). Consequently, (i) the presence of word comprehension impairments had very low sensitivity to impairments on any of the other tasks, even for patients with the most severe word comprehension impairments; and (ii) word comprehension impairments were only weakly associated with impairments on other tasks (~ 25%) regardless of the severity of impairment on the other tasks. Nevertheless, as expected given the greater dependence of sentence comprehension on word comprehension, when word comprehension impairments were present, they co-occurred more frequently with sentence comprehension impairments (84%) than with sentence repetition impairments (71%).

We discuss these observations below offering explanations for the influence of aphasia severity and addressing sources of variability in incidence, PPV and sensitivity that arise (i) between studies, (ii) within study, and (iii) from sampling bias. We also discuss the underlying neurocognitive mechanisms that determine aphasia profiles, and the broader clinical and theoretical and future relevance of our findings.

### Explaining the influence of impairment severity

We offer three potential explanations for why incidence, PPV and sensitivity are far lower when language impairments are less severe. The first is that all but one of the patients with selective impairments (i.e., on one task only) had less severe impairments. By definition, aphasia in these patients can only be detected by directly testing them on the tasks they are impaired on. Consequently, performance on other language tasks is necessarily insensitive to selective impairments, because impairment does not generalise beyond the affected domain.

The second possibility is that the least selective impairments sometimes reflect patient errors driven by stroke-related factors such as fatigue, poor attention or concentration which are common after stroke, not neccessarily related to aphasia^47–49^ and fluctuate across the task selection. Multiple assessments would help control for these errors.

The third explanation relates to inherent uncertainty as to whether a score bordering the aphasic cut-off value reflects pre-stroke ability or stroke-related impairment. In all standardised aphasia assessments, aphasic cut-off scores are determined, for each task separately, based on population-level data. This is necessary because, in the absence of pre-stroke scores, aphasia cannot be diagnosed by comparing post-stroke to pre-stroke performance. However, this approach cannot determine whether language scores just below the aphasic cut-off are a consequence of low pre-stroke ability or post-stroke aphasia. Consequently, healthy neurotypical individuals with lower verbal abilities (e.g. vocabulary) which is known to be associated with lower performance on language tasks^60^, may score in the 5th percentile of normal which, by definition, is within the “aphasic” range even though low performance is not a consequence of stroke.

Given these possibilities, it becomes clear that patients with the least severe impairments require more detailed follow-up assessments to accurately identify the underlying factors that explain their language performance. One useful approach is to ask the patients, or their families, for their own perspectives on how their abilities have changed post-stroke. More broadly, it is good clinical practice, when evaluating language function, to integrate patient and carer reports with formal assessment results and other relevant clinical information.

### Between study variability in the incidence of language impairments

The incidence of naming and word comprehension impairments in the current study (66% and 20%) were notably lower than in Grönberg et al.^27 ^(79% and 41%) with negligible inter-study differences in sentence comprehension (67% vs. 64%), and word and sentence repetition, (58–59% vs. 62%). Richardson et al.^26^ also reported a higher incidence of naming impairments (82%) than the current study (66%) but is not directly comparable because it used a broad definition of aphasia from the WAB that includes impairments at the level of spontaneous speech (e.g. Information content and Fluency), and functional word finding (e.g. sentence completion and conversational responses) in addition to repetition and comprehension. Because these additional impairments draw on cognitive and functional processes that overlap with confrontation naming, the co-occurrence of anomia is likely to be higher than when the aphasic cohort is more narrowly defined by repetition, comprehension or naming impairments only.

Below we consider five possible explanations for why we found a lower incidence of naming and word comprehension impairments than Grönberg et al.^27^: time post-stroke at assessment, choice of aphasia assessment, definition of aphasic performance, cohort size and participant inclusion criteria.

**Time post-stroke at assessment** was months to years in the current study but only a few days in Grönberg et al.^27^. Plausibly, patients in the current study had more time to recover. Indeed, some evidence suggests that anomia may recover faster than other impairments^61^, and substantial improvement in auditory comprehension often occurs within the first three to four months^62–64^, or even as early as two weeks after stroke^65^. Aphasia profiles also evolve from non-fluent to fluent within the first year of recovery^38,39,61^. Together these findings motivate longitudinal studies following the same individuals from the acute to the chronic phase to determine how recovery trajectories shape aphasia profiles at different time points.

**The choice of naming assessment** introduces differences in stimuli and task instructions that can influence the diagnosis of anomia. Participants named 24 objects in the current study but only five in Grönberg et al.^27^. The proportion of objects with low frequency names is therefore likely to have varied across studies. Even for the same objects, there could be inter-study differences in the stimuli (e.g. drawings) which influence familiarity and naming accuracy independently of language impairment. Updating visual materials to reflect contemporary vocabulary and cultural norms could improve test validity. A range of other assessment factors may also affect performance. For example, the CAT naming subtest was developed to account for several key psycholinguistic variables known to affect naming performance, including word length, frequency, morphological complexity, regularity, imageability, and animacy. However, other variables, known to affect naming^53,54^ are not explicitly controlled in the CAT, such as age of acquisition, phonetic (or phonological) complexity, semantic category, and prototypicality.

**The definition of aphasic performance** in the CAT was based on a small normative reference sample (*n* = 27) that differed in sex distribution from the patient cohort, potentially affecting the precision of aphasic cut-offs. A small normative dataset may fail to capture the wide normal inter-individual variability in language performance due to factors such as age^66–70^, vocabulary knowledge^60^, education^71^, cultural background, and dialect^72^, which may consequently overestimate or underestimate aphasia incidence. Parallel validation against independent assessments such as the Western Aphasia Battery [WAB^8^] or the Boston Diagnostic Aphasia Examination [BDAE^7^] would strengthen diagnostic reliability.

**Cohort size** was 382 individuals with chronic post-stroke aphasia in the current study which is more than 6 times bigger than the sample of 58 participants in the Grönberg et al.^27^ study. Larger samples, however, do not necessarily yield more stable estimates if the participants included increase heterogeneity in demographic composition, stroke aetiology, lesion site, and time post-stroke. Such heterogeneity can introduce genuine variability in performance profiles that is independent of measurement error. Future cross-cohort comparisons and meta-analytic approaches should therefore account for these factors, without assuming that increased sample size alone reduces statistical variability.

**Participant Inclusion criteria** is a fifth source of inter-study differences. We excluded patients with impaired vision, hearing and object recognition (assessed with the CAT semantic association picture matching task). If one task is affected by these variables more than another, then the relative incidence of impairments across task could change. That said, we didn’t find evidence of this because the inclusion of 49 patients with semantic matching impairments increased the incidence of both naming (66% to 69%) and sentence comprehension (67% to 70%) but the incidence of naming impairments was still lower than that reported in Grönberg et al. (79%).

### Within study variability in Incidence PPV and sensitivity

Within-study variability can arise from task selection and measurement error. We found that when additional tasks such as action naming or reading were included, there were notable differences in the incidence and predictive value. For instance, defining anomia as an impairment in either object or action naming increased its incidence from 66% to 80% but reduced its PPV from 90% to 78%, whereas adding reading decreased anomia incidence to 58% but slightly increased its PPV to 93%. These shifts confirm that incidence and PPV are not fixed properties of a test or symptom but depend critically on the specific set of language functions under consideration.

Measurement variability within study, irrespective of the tasks used, is illustrated by examining the reasons for our observation that 12 of the 76 patients with aphasic word comprehension were *not* diagnosed with aphasic sentence comprehension, despite the dependence of sentence comprehension on word comprehension. We identify five potential factors (A-E) that may account for this dissociation: (A) Of the 12 patients who scored in the aphasic range on word comprehension but not sentence comprehension, all but 3 had the highest possible aphasic score in word comprehension (raw score = 25/30, T-score = 51 = the aphasia cut-off score) leaving it uncertain as to whether their score truly reflected aphasia (see above). (B) The word stimuli used in the sentence comprehension subtest were not identical to those in the word comprehension subtest and may have been more familiar to some patients, even if the sets were fully matched on key psycholinguistic variables across tasks. In addition, some patients may have found the word stimuli more lexically or semantically demanding resulting in disproportionate difficulty for these particular patients. (C) Brief lapses of attention may have transiently lowered performance during the word comprehension task but not the sentence comprehension task. (D) Some patients may be able to compensate for partial lexical deficits by drawing upon syntactic or contextual cues in the sentence task. (E) Differences in task format or response demands between the subtests could further modulate apparent comprehension ability. For example, for some patients, the pictures used in word comprehension stimuli might be less recognisable than those used in sentence comprehension.

In summary, irrespective of the aphasia tests employed, apparent dissociations between tasks can arise from cumulative sources of inter-patient and measurement variability, highlighting the need for cautious interpretation of classic diagnostic metrics. Future work could incorporate independent assessments of aphasia to strengthen diagnostic validity and reduce potential inflation of selective impairment frequencies. For example, parallel validation could involve comparing CAT-based classifications with outcomes from other established language assessments such as the Western Aphasia Battery [WAB^8^] or the Boston Diagnostic Aphasia Examination [BDAE^7^]. Alternatively, cross-battery validation could draw on large-scale datasets in which different aphasia tests have been used, to examine whether similar incidence rates and impairment profiles emerge across test batteries. Such approaches would help determine whether observed dissociations reflect true cognitive differences or task-specific variability, thereby improving the robustness and interpretability of incidence estimates.

### Sampling bias in research studies

Studies like the current one that analyse impairment profiles across a sample of aphasic participants are invariably subject to sampling bias. Specifically, participants who volunteer to take part in research are likely to be healthy, well-educated and from comfortable socioeconomic backgrounds^73,74^. They may therefore perform better in standardised testing than patients who do not volunteer for research. Future studies would need to investigate whether aphasia profiles are affected by these factors. Research participants are also less likely to have the most severe comprehension impairments as this makes it more difficult for them to consent to the study or understand the assessment instructions. This may partly account for why impaired word comprehension (the most severe form of aphasia) was the least observed impairment in the current study. This recruitment barrier can be addressed by obtaining consent from participants through their consultees, and supplying training materials to recruitment staff to equip them with the skills to effectively communicate and consent stroke survivors who have severe (word) comprehension difficulties.

### Neural and cognitive explanations

The strong co-occurrence of the most severe naming, repetition, and comprehension impairments likely reflects shared underlying neural damage. Such overlap may arise from focal injury to cognitive systems common across language tasks, such as (i) phonological production required for both naming and repetition^75,76^, (ii) verbal short-term memory supporting naming and comprehension^77–80^, and/or (iii) articulatory planning and co-ordination needed for speech motor control in naming and repetition^22,81,82^. Alternatively, it may result from widespread damage that disrupts susceptible vascular territories and multiple neural systems involved in different aspects of language^83,84^. Consistent with the latter, patients with the most severe anomia had mean lesion volumes of 172 cm³ which is approximately double those of patients with the least severe anomia (66 cm³).

Future studies could disentangle these mechanisms by identifying which neural systems support co-occurring versus selective aphasic symptoms. This can be achieved by combining detailed behavioural testing with neuroimaging data, as in many recent studies^85–90^ that systematically assessed lesion sites associated with lexical retrieval, semantic processing, phonological encoding, articulatory planning, and motor execution. More specifically, a neuroimaging study of the current data (or similar sample) could directly compare lesion location in groups of patients with (i) co-occurring naming and repetition impairments, (ii) co-occurring naming and comprehension impairments, (iii) selective naming impairments, (iv) selective repetition impairments and (v) selective comprehension impairments. Ultimately, such work could enable a more biologically grounded classification of aphasia, linking behavioural profiles to underlying cognitive and neural architecture rather than relying solely on descriptive symptom clusters.

### Clinical and theoretical implications

These findings temper long-standing assumptions that naming is the hallmark or most diagnostically informative feature of aphasia. While anomia may be the most salient symptom of aphasia, and remains a key clinical indicator, its incidence, PPV and sensitivity was statistically equivalent to that of sentence comprehension and the repetition tasks. The clear exception was word comprehension, because the low incidence of impairments on this task constrained the likelihood that these impairments would co-occur with other more frequent impairments. Consequently, a multi-task assessment remains essential for capturing the full heterogeneity of aphasic profiles. Reliance on naming alone, or even a subset of tasks, would likely fail to detect mild or selective deficits in repetition or comprehension, which may still have functional consequences for communication. What we can deduce from one task only, is that the absence of the most severe impairments makes it highly unlikely that the most severe impairments will be observed on any other of the tasks we test in this study.

As post-stroke anomia is such a painful source of frustration for patients and their loved ones^91^, it is essential that its root cause is identified and targeted directly, in order to maximize recovery outcomes. By investigating how different language impairments co-occur and dissociate depending on severity and task, the processing impairments underlying aphasic symptoms can be identified. Developing better diagnostic metrics will in turn help to improve prognosis and treatment planning for individuals with aphasia.

## Summary and conclusions

In summary, when using the Comprehensive Aphasia Test battery^11^, in a large sample of aphasic patients in the chronic phase of post-stroke recovery, object naming was no more diagnostically informative than other core language tasks, with the exception of word comprehension which displayed strikingly lower sensitivity due to the low incidence of such impairments. We argue that, given the presence of selective impairments in each of the language tasks tested, diagnosis of aphasia requires a multi-task assessment.

Our study shows that patterns of co-occurrence among language tasks depend jointly on the reference task, the other impairments being evaluated, participant selection, impairment severity, the incidence of selective impairments, the sample being studied and likely (but not yet proven) the time post-stroke that they are assessed. Diagnostic-style metrics should therefore be interpreted with caution and findings replicated across other assessment batteries.

New directions for this work include longitudinal studies to trace how co-occurrence of impairment patterns evolve over time from the acute post-stroke stages to later stages of recovery. Combining behavioural, neuroimaging, and computational approaches will also help to link distinct processing deficits with neural substrates.

## Data Availability

The data supporting the findings presented in the current study can be requested from the corresponding author (S.A.).
